# Complete response after autologous stem cell transplant in multiple myeloma

**DOI:** 10.1002/cam4.257

**Published:** 2014-04-29

**Authors:** Lalit Kumar, Nida Iqbal, Anjali Mookerjee, Rakesh Kumar Verma, Om D Sharma, Atul Batra, Raja Pramanik, Ritu Gupta

**Affiliations:** Department of Medical Oncology and Laboratory Oncology Institute Rotary Cancer Hospital, All India Institute of Medical SciencesNew Delhi, 11 00 29, India

**Keywords:** Autologous stem cell transplant, complete response, multiple myeloma, pretransplant therapy

## Abstract

We evaluated long-term outcome of patients achieving complete response (CR) after autologous stem cell transplantation (ASCT) for multiple myeloma. Between April 1990 and June 2012 191 patients underwent ASCT. The median age was 53 years (range, 26–68 years), 135 were men. Pretransplant, patients received induction therapy with VAD (vincristine, doxorubicin, dexamethasone; *n* = 77), novel agents (*n* = 92), or alkylating agent-based, *n* = 22); 43% received more than one line of induction regimen. Response to transplant was defined as per EBMT criteria. The median follow-up for the entire group was 85 months (range, 6–232.5 months). Following transplant 109 (57.1%) patients achieved CR. Median progression-free survival (PFS) for patients with CR was higher compared to those with VGPR and PR, (107 vs. 18 vs. 18 months, *P* < 0.001). Number of lines of therapy pretransplant (one or two vs. more than two lines of therapy (*P* < 0.001), and absolute lymphocyte count of ≤3000/cmm were predictors of superior PFS. Median overall survival (OS) for patients with CR was higher, (204 months), compared to those with VGPR (71.5 months, *P* < 0.001) and PR (51.5 months, *P* < 0.001), respectively. On Cox regression analysis, patients who received one line of induction therapy pretransplant (hazard ratio, HR 2.154, *P* < 0.001) and those with absolute lymphocyte count of ≤3000/mm^3^ (HR 0.132, *P* < 0.001) had superior PFS. For overall survival, induction treatment up to one line (HR 2.403, *P* < 0.004) and Hb > 7.1 G/dL at diagnosis (HR 4.756, *P* < 0.01) were associated with superior outcome. On landmark analysis at 12 months, PFS and OS continued to remain superior for patients attaining CR. Achievement of CR post transplant is associated with longer OS and PFS. Among complete responders, those who receive one line of induction therapy pretransplant have superior outcome.

## Introduction

High-dose chemotherapy followed by autologous stem cell transplantation (ASCT) is considered a standard treatment approach for patients of multiple myeloma (MM) aged 65 years or younger 1,2. Achievement of complete response (CR) post transplant is an important goal and is associated with longer progression-free survival (PFS) and usually better overall survival 3–7. The duration of PFS varies from 18 months to more than 60 months, reflecting variable amount of minimal residual disease or depth of CR. A small proportion achieves long-term progression-free survival and can be considered to be operationally cured 8,9. Identification of complete responders likely to have shorter PFS post transplant may help to adapt alternative strategies other than standard maintenance therapy currently being followed in the management of such patients. We analyzed the data of 191 patients of myeloma who underwent ASCT at our center; 109 of them achieved CR post transplant. These are the subjects of this report.

## Patients and Methods

Between April 1990 and June 2012 191 patients with MM underwent ASCT. Patients' age ranged from 26 to 68 years (median 53 years). There were 135 male and 46 female patients. The database was maintained prospectively. Before transplant, patients had received induction therapy either using VAD (vincristine, doxorubicin, dexamethasone; *n* = 77), novel agents (thalidomide and dexamethasone, or lenalidomide and dexamethasone, or bortezomib and dexamethasone; *n* = 92), or alkylating agents (VMCP [vincristine, melphalan, cyclophosphamide, and prednisolone] or MP [melphalan and prednisolone], *n* = 22); 43% of patients received more than one line of induction regimen. Overall, 140 (73.3%) had chemo-sensitive disease (including complete response [CR], very good partial response [VGPR], and partial response [PR]) before ASCT. Of these, 44 patients (23%) had renal insufficiency at diagnosis and 16 (8.3%) had renal dysfunction at the time of transplant. Baseline characteristics for all patients and in different post transplant response categories are shown in Table1.

**Table 1:** Baseline characteristics at diagnosis according to transplant response.

	Total	CR	VGPR	PR	Stable	*P*-value
No. of Patients	191	109 (57.1%)	33 (17%)	25 (13.1%)	8 (4.2%)	
Gender, M:F	135:56	79:30	23:10	20:5	4:4	0.11
DSS stage (*n* = 191), ≤3A vs. 3B	147:44	89:20	26:7	22:03	6:2	0.98
ISS (*n* = 188), I/II/III	71:72:45	45:41:23	11:17:04	13:07:04	2:01:04	0.01
Ig type, IgG/IgA/light chains	135:23:30	56:14:15	22:3:5	18:1:1	7:0:0	0.24
Serum M protein, median (range)	2.97 (1–7.57)	3.46	3.5 (1–6.79)	3.3 (0.36–7.1)	2.50 (0.78–10.0)	0.99
Hb (G/dL), mean (range)	9.9 (3.2–16.0)	10.0 (3.2-15.5)	10 (5.4–17.7)	9.6 (5.9–12.8)	9.7 (6.6–12.5)	0.70
Albumin (G/dL), median (range)	3.80 (1.8–5.70)	3.8 (1.8–5.7)	3.6 (2.30–)	3.8 (2.7–5.2)	3.30 (2.5–3.9)	0.25
BM plasma cell%, mean (range)	49.5 (1–100)	51.8 (1–100)	40 (2–85)	36.7 (2–90)	56.5 (10–100)	0.5
Serum B2M, median (range)	3.041 (1116–325,518)	3.07 (1116–32,625)	3434 (1310–8067)	2500 (1279–8008)	4381 (1698–8008)	0.95
Median ALC (range), at diagnosis	2402 (230–21,960)	1923 (9288–21,960)	2195 (230–3401)	2552 (528–8432)	2082 (1606–5244)	0.25
Period of study, 2001/2001–2005/2006–11	40:52:99	15:24:70	7:15:11	10:7:8	2:2:4	0.004
Median interval from diagnosis to Tx in months, (range)	10 (2–128)	9 (4–128)	12 (2–48)	11 (3–66)	32 (4–61)	0.05
Treatment Novel agents/VAD/alkylating agents	92:77:22	67:37:05	8:22:03	8:11:6	3:3:2	0.001
Induction therapy: one line/two lines/more than two lines	109:56:24	76:22:11	15:13:04	12:8:5	2:3:2	0.001

VAD, vincristine, adriamycin, dexamethasone; ALC, absolute lymphocyte count; CR, complete response; VGPR, very good partial response; PR, partial response; BM, bone marrow.

### Transplant protocol

Details of transplant protocol, initial results, and supportive care have been described earlier 10,11. The source of stem cell was bone marrow in the first seven patients; for the next 184 patients granulocyte colony-stimulating factor (G-CSF) mobilized peripheral blood stem cells were harvested. Stem cells were transfused intravenously 24 h after high-dose melphalan (200 mg/m^2^). Patients with renal insufficiency at the time of transplant received reduced dose of melphalan (120–150 mg/m^2^). Stem cells were re-infused on day 0 through a central venous catheter (Hickman) preceded by pheniramine maleate 50 mg i.v. Post stem cell infusion patients received G-CSF 5 mcg/kg daily subcutaneously on day +1 onward until engraftment. Once engrafted and stable, patients were discharged and were followed up on an outpatient's basis. Response to transplant was assessed 6 weeks after transplant on two occasions (day 100) as per European Group for Blood and Bone Marrow Transplantation (EBMT) criteria 12.

### Post transplant maintenance therapy

Until December 2001, patients received maintenance therapy, with interferon-alfa at a dose of 3 million units thrice a week subcutaneously. From January 2002 onward, all responding patients received thalidomide 50 mg daily for 1 year or more. Maintenance therapy was initiated when engraftment was stable (absolute neutrophil count—2000/mm^3^, platelets ≥ 100,000/mm^3^). Therapy was continued for 12 months or more. Patients also received zoledronic acid 4 mg i.v. once in a month for 6–9 months since diagnosis then once in 3 months for the initial 2 years then once in 4–6 months indefinitely.

### Statistical analysis

Analysis has been done as intent-to-treat analysis. Descriptive statistics (median and range) were calculated for all variables. Response to transplant was defined as per EBMT criteria 12. Duration of complete response was defined as time from achievement of CR to relapse. The prognostic factors for response to transplant were analyzed by Pearson chi-square test and binary logistic regression analysis. Overall survival was defined as the time from date of transplant until death or date of censor (30 December 2012). Progression-free survival (PFS) was calculated from date of transplant to disease progression or death (regardless of cause of death). Survival curves were plotted according to method of Kaplan and Meier and were compared by the log-rank test. Land mark analysis was done at 12 months. The prognostic factors for survival were analyzed by Cox regression analysis. Analysis was carried out using SPSS-16 statistical software (IBM, Armonk, NY). The median follow-up for the whole group is 85 months (range, 6–232.5 months).

## Results

### Patients' characteristics

Table1 shows patients' characteristics at diagnosis according to transplant response. Patients' characteristics were similar according to age, gender, renal functions, myeloma subtype, hemoglobin (G/dL), serum albumin, bone marrow plasma cell percentage, year of treatment, and absolute lymphocyte counts at diagnosis. The median interval from diagnosis to transplant was shorter for patients in CR compared to those in VGPR, PR, and stable response disease (9 vs. 12 vs. 11 and 32 months <0.05). Higher proportion of patients received novel agents in CR category compared to those in VGPR, PR, and stable response category (61.5%, vs. 24.2%, vs. 32% and 37.5%, *P* < 0.001). Similarly, higher proportion of patients had received one line induction therapy in CR category (69.7%) compared to those in VGPR, PR, and stable response category (46.7%, 48% and 25%, *P* < 0.001). Higher number of patients had ISS III in Stable response category (57%) compared to those with CR, VGPR, and PR response (21%, 12.5%, 16.6%, respectively, *P* < 0.01. (Table1).

### Response to transplant

Post transplant response evaluation (day 100) revealed: complete response: 109 (57.1%), VGPR-33 (17.3%), partial response 25 (13.1%), and stable disease in 8 (4.2%) patients. Among 109 patients with CR—35 patients (32.1%) were in CR Pretransplant, these continued to remain in CR post transplant. Of the remaining 74 patients—35 (32.1%) had VGPR, 30 (27.5%) partial response, and 8 (7.3%) stable disease and one had progressive disease, these were induced in CR post transplant.

### Survival according to transplant response

Median PFS for patients with CR was 107 months which was significantly better compared to those with VGPR and PR, (*P* < 0.001). There was no difference in PFS for patients who achieved VGPR and PR. Median overall survival (OS) for patients with CR is 204 months, which is higher compared to those with VGPR and PR (*P* < 0.001 and <0.001). There was no difference in overall survival between VGPR and partial responders (*P* = 0.43) (Table2).

**Table 2:** Survival according to response to transplant.

Post transplant response	No. of Pts	Progression-free survival (months)		Overall survival (months)	
		Median	95% CI	Median	95% CI
CR	109	107	51.1–162.9	204.0	108.5–299.4
VGPR	33	18	16.0–20.0	71.5	24.07–118.9
PR	25	18	14.8–21.2	51.5	25.1–77.9
Stable	8	6.0	3.2–8.7	11.0	6.8–15.2
Total	175	34.0	21.2–46.8	125	82.9–168.1
	CR vs. VGPR, *P* < 0.001			CR vs. VGPR, *P* < 0.001	
	CR vs. PR, *P* < 0.001			CR vs. PR, *P* < 0.001	
	CR vs. stable response, *P* < 0.001			VGPR vs. PR, *P* = 0.43	
	VGPR vs. PR, *P* = 0.86			PR vs. stable, *P* < 0.04	
	PR vs. stable response, *P* < 0.001				

CR, complete response; VGPR, very good partial response; PR, partial response.

#### Land mark analysis at 12 months

**Figure 1:**
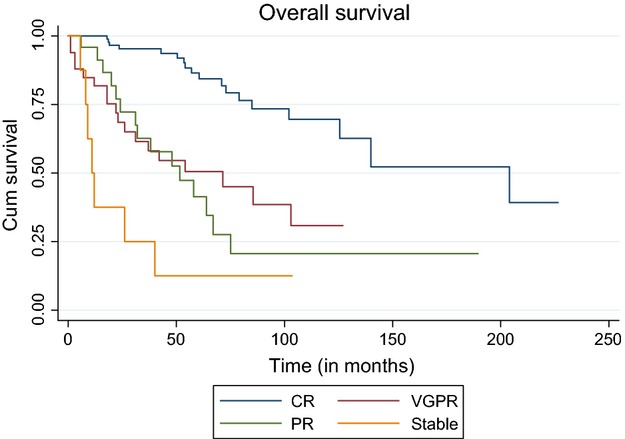
Overall survival according to response to transplant.

**Figure 2:**
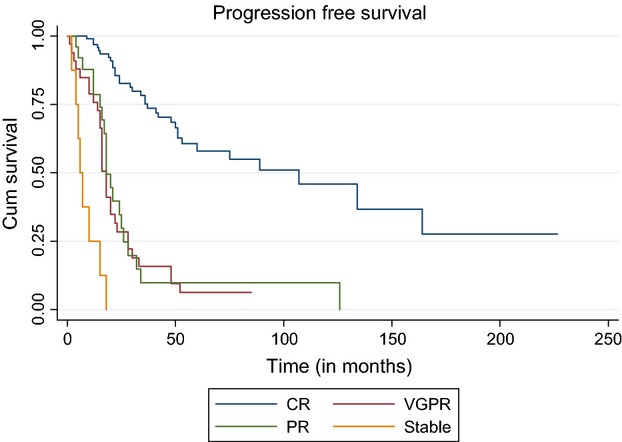
Progression-free survival according to response to transplant.

We performed land mark analysis for overall survival and PFS at 1 year. At 12 months, 95 patients were alive in CR category, VGPR—27, PR—21, and 3 in the stable response category. For patients with CR—median OS was 204 months (95% CI 108.5–299.6). This was significantly higher compared to VGPR (median 85.5 months (95% CI 42.8–128.2) and partial response (median 51.5 months (95% CI 25.5–77.5), *P* < 0.001. Median PFS for CR category was 107 months (95%CI 51.04–162.9), this was significantly higher compared to those with VGPR- 20 months (95% CI 16.1–23.9) and PR-20 months (95% CI 12.8–29.2), *P* < 0.001 (Figs.1 and 2).

For complete responders estimated PFS at 5, 10, and 15 years is 70.3% ± 0.05 (SE), 45.9% ± 0.08 (SE), and 27.5% ± 0.11(SE), respectively. Corresponding overall survival is 79.3% ± 0.05 (SE), 69.6% ± 0.07 (SE), and 39.2% ± 0.14(SE) at 5, 10, and 15 years, respectively.

#### Pretransplant CR versus induced CR

For patients in CR pretransplant—median PFS was 60 months (95% CI 20.6–99.5 compared to 107 months (95% CI 35.8–178.2), for those with post transplant CR (induced CR), *P* = 0.94. Median overall survival is 140.0 months versus and 204.0 months for those with pretransplant CR and induced CR, respectively, *P* = 0.81.

### Prognostic factors

#### Progression-free survival

Number of lines of therapy (one or two vs. more than two lines of therapy) prior to transplant, absolute lymphocyte count (absolute lymphocyte count 3000/cmm or less at diagnosis, were important predictors of superior progression-free survival (Table3).

**Table 3:** Complete response: progression-free and overall survival—analysis of prognostic factors: univariate analysis.

	Patient no.	Median PFS (months)	95% CI	*P*-value	Median OS (months)	95% CI	*P*-value
Age							
≤53 years vs.	53 vs.	89 vs.	38.6–139.4	0.70	204 vs.	121.6–286.4	0.23
>53 years	56	134	0–276.1		125.5	–	
Gender							
Male vs.	79 vs.	107 vs.	56.2–157.8	0.66	140	62.6–217.4	0.13
Females	30	75	26.9–123.0		NR	–	
Interval: diagnosis toTx							
≤12 months vs.	77 vs.	134 vs.	25.6–242.4	0.18	–	–	0.04
>12 months	32	89	24.1–153.9		204	–	
≤9 vs.	63 vs.	NR vs.	–		NR vs.	–	
>9 months	46	89	9.6–168.4	0.10	204.0	58.7–349.2	0.11
Interval -diagnosis to Tx For novel agents only							
≤12 months vs.	46	NR	–	0.15	NR	–	0.07
>12 months	21	NR	–		NR		
≤9 vs. >	38	NR	–	0.16	NR	–	0.22
9 months	29	NR			NR	–	
ISS							
I+II	86	107	59.8–154.1	0.79	–	–	
III	23	164	33.9–294.1		204	108.5–299.5	0.66
DSS							
≤IIIA	89	134	24.6–243.4	0.15	204	62.6–345.4	0.21
IIIB	20	60	33.3–86.7		79	19.5–138.5	
Ig type (*n* = 108)							
IgG	71	164	29.9–298.1	0.16	204	90.3–317.7	0.66
IgA	17	51	32.6–69.4		140	35.23-244.7	
Light chains	20	53	5.7–100.3		NR	–	
Serum albumin							
≤3.5 G/dL	39	60	0–129.8		140	110.4–169.5	
>3.5 G/dL	70	89	52.2–125.8	0.57	NR	NR	0.66
≤3.4 G/dL	31	51	36.9–65.1	0.21	125.5	67.4–183.6	0.26
>3.4 G/dL	78	107	60.2–153.8		NR	–	
Plasma cell% (*n* = 107)							
≤40%	60	107	62.1–151.8	0.83	204	89.7–318.3	0.61
>40%	47	60	–		140.0	–	
Absolute lymphocyte count at diagnosis (*n* = 84)							
≤3000/cmm	71	107	62.4–151.6	0.001	140	53.7–226.3	0.19
>3000/cmm	13	19	10.5–27.5		–	–	
≤2500/cmm	60	107	26.2–187.8	0.04	140	–	
>2500/cmm	24	34	–		–	–	
Induction therapy							
Novel agents	67	NR	–		NR	–	0.59
VAD	37	75	2.5–147.4	0.91	204	105.2–302.8	0.90
Alkylating agents	05	89	0–183.5		NR	–	
No. of regimen (s) pretransplant							
One line	76	164	62.7–265.3		204	–	
More than one line	33	41	11.8–70.2	0.001	85	29.0–140.9	0.01
2 lines	98	107	54.9–159.0		204	64.5–343.5	
More than 2 lines	11	24.0	21.7–26.3	0.004	71	–	0.006
Hb (G/dL) (*n* = 108)							
≤10	56	134.0	69.7–198.3	0.38	140	–	0.60
>10	52	53.0	10.2–95.8		204	89.85–318.1	
≤7	14	37.0	20.8–53.2	0.11	79.0	43.4–114.6	0.01
>7	94	134.0	73.7–194.3		204.0	82.2–325.8	

VAD, vincristine, adriamycin, dexamethasone.

#### Overall survival

Presence of severe anemia at diagnosis (≤7 G/dL), and more than one or two lines of induction therapy were associated with inferior overall survival. (Table3).

1. Cox regression analysis progression-free survival: patients who had received one line of therapy prior to transplant and those who had absolute lymphocyte count ≤3000/cmm had superior PFS.
2. Overall survival: pretransplant—one line of therapy, and Hb > 7.1 G/dL were predictors of better overall survival (Table4).

**Table 4:** Cox regression analysis.

Factor	HR	95% CI	*P*-value
Progression-free survival			
Pretransplant: one line therapy	2.154	1.422–3.263	<0.001
ALC ≤ 3000/cmm vs. >3001/cmm	0.132	0.053–0.327	<0.001
Overall survival			
Pretransplant: one line therapy	2.403	1.325–4.358	<0.004
Hb (G/dL) ≤7.1 vs. >7.1	4.756	1.357–16.668	<0.015

ALC, absolute lymphocyte count; HR, hazard ratio.

### Current Status

Among 109 patients, 21 have died: causes include—18 due to progressive disease and associated complications (plasma cell leukemia-5, chest infection-3, renal failure-1, sepsis with multi-organ failure-1, myocardial infarction-1). One patient died of secondary leukemia, another due to viral illness and in one patient cause of death was not known. Eighty-eight patients are currently alive; 30 (27.5%) with disease and are on salvage therapy, remaining 58 (53.2%) patients continue to be alive and disease free.

## Discussion

Achievement of CR is an important event in myeloma and represents the major surrogate marker for long-term PFS and OS. In this study, 57.1% of patients achieved CR post transplant. Median progression-free and overall survival of patients with CR is 107 months and 204 months, respectively. This was higher than those with VGPR, and partial response. Patients who received only one or two lines of therapy pretransplant and those with Hb > 7.1 G/dL at diagnosis had significantly better outcome.

CR rates to transplant in this study are higher than those reported in earlier studies using only chemotherapy-based induction prior to transplant 1. These rates are similar to studies reported after year 2000 where novel agent- based induction therapy (immune-modulatory drugs—thalidomide, lenalidomide, and proteasome inhibitor bortezomib) have been used 4–6,8. We made an attempt to identify baseline characteristics between different response categories (Table1). Patients in CR category had received transplant earlier (median interval from diagnosis to transplant was shorter, *P* < 0.05), higher proportion of patients had ISS stage I, more patients received novel agents, *P* < 0.001) and higher proportion of patients had received one line therapy prior to transplant <0.001). Higher number of patients in Stable response category had ISS III stage, *P* < 0.02. Significant variation in many baseline clinical and laboratory characteristics among patients with different response category reflects the heterogeneity of multiple myeloma.

Achievement of CR post transplant is related to pre-transplant disease status. This is supported by our observation that among 140 patients with pretransplant chemo-sensitive disease, 100 (71.4%) achieved CR compared with 9 of 51 (17.4%) with stable or progressive disease pretransplant.

In this study, PFS and OS were better for complete responders compared to those achieving VGPR or PR. A number of investigators have confirmed positive association between achievement of CR and improved progression-free and overall survival 4–6,8,13,14. Harousseau et al. for the French Group identified achievement of “at least VGPR” as an important predictor of outcome in an analysis of 802 patients 15. Association of CR with superior outcome has also been reported in non transplant setting also 16,17. We did not find difference in OS and PFS for 37 patients who were in CR pretransplant, 35 of 37 continued to be in CR post transplant versus 74 patients who achieved CR post transplant (induced CR from pretransplant VGPR, PR, or stable disease status) as reported earlier 18.

When should transplantation in myeloma be done has been a subject of debate. In this study, there was a favorable trend for median PFS for patients with CR who underwent ASCT within 9 months (median not reached vs. 89 months, *P* = 0.10) or 12 months (134 vs. 89 months, *P* = 0.18 of diagnosis (Table3).

Sixty-seven of 109 complete responders had received novel agent-based induction therapy; median has not reached in either group, there was a favorable trend for mean PFS for those transplanted within 12 months—66 months ± 4.5 (SE) (95% CI 55.2–74.8) versus 39.8 months ± 4.7 (SE) (95% CI 30.5–49.0), *P* = 0.15 and also for those transplanted within 9 months; mean PFS 66.9 ± 4.7 (SE) (95% CI 57.8–76.0) versus 40.6 months ± 4.1 (SE) (95% CI 32.6–48.6), *P* = 0.16. Similar trend was seen for overall survival too. For those transplanted within 12 months; mean Overall survival was 78.3 months ± 3.1(SE) (95% CI 72.1–84.5) versus 52.0 months ± 3.6 (SE) (95% CI 44.8–59.1), *P* < 0.07. For those transplanted within 9 months; mean OS was 77.6 months ± 3.5 (95% CI 70.7–84.6) versus 53.5 months ± 2.9 (95% CI 47.7–59.3), *P* = 0.22. Fermand et al. in a randomized French study reported results of 200 patients who underwent ASCT either immediately after induction therapy or after they had relapsed; patients who received transplant earlier had a higher median PFS compared with those who received transplant later (39 vs. 13 months, *P < *0.01) but overall survival was not significantly different 19.

In this study, 76 of 109 patients had received one line of therapy prior to transplant; PFS was significantly higher for this group of patients compared to those who had received more than one line of therapy; 164 versus 33 months <0.001. Similarly, patients who had received up to two lines of therapy (*n* = 98) had a better PFS; 107 versus 24.0 months <0.004. Similar effect was seen for overall survival too, both for one line (*P* < 0.01) and up to two lines of therapy (*P* < 0.006) pretransplant (Table3). These observations and also of Lahuerta et al. for the Spanish Myeloma Group 4 suggest that treatment beyond two lines of therapy (to convert partial responders to VGPR or CR status) pretransplant may not help or rather may be counterproductive. Implications of this observation could be that one needs to identify (restage) patients with suboptimal response midway (60–90 days) rather continuing therapy for four to five cycles which is currently the practice. Furthermore, patients who have received more than two regimens pre-transplant and have achieved CR following transplant may be considered for more aggressive maintenance therapy.

We used EBMT criteria 12 to define response (CR defined by immunofixation). Recent studies have used more stringent criteria defined by International Myeloma Working Group 20. Monitoring of CR patients for minimal residual disease using more sensitive techniques such as quantitative reverse transcription polymerase chain reaction (RT PCR) and/or flow cytometry may help to identify patients at high risk of relapse 21–23. Such patients with persistent disease or rising amount of monoclonal proteins could be considered for more aggressive maintenance or short sequential therapies to prevent relapse. Lack of cytogenetic data is a major limitation of this study. Such information and comprehensive gene expression profiling has helped to identify high-risk patients 24 with possibility to develop risk-adapted strategies.

## Conclusion

This study confirms that high-dose chemotherapy supported by autologous peripheral blood stem cell transplant in patients with advanced myeloma is associated with a high CR rate. Patients who achieve CR post transplant have longer OS and PFS. Use of up to one line of induction therapy is associated with better outcome.
